# A Unique Robust Dual-Promoter-Driven and Dual-Reporter-Expressing SARS-CoV-2 Replicon: Construction and Characterization

**DOI:** 10.3390/v14050974

**Published:** 2022-05-05

**Authors:** Ying Liu, Lu Li, Khalid A. Timani, Johnny J. He

**Affiliations:** 1 Department of Microbiology and Immunology, Chicago Medical School, Rosalind Franklin University, North Chicago, IL 60064, USA; ying.liu@rosalindfranklin.edu (Y.L.); lu.li@rosalindfranklin.edu (L.L.); khalid.timani@rosalindfranklin.edu (K.A.T.); 2 Center for Cancer Cell Biology, Immunology and Infection, Rosalind Franklin University, North Chicago, IL 60064, USA; 3 School of Graduate and Postdoctoral Studies, Rosalind Franklin University, North Chicago, IL 60064, USA

**Keywords:** SARS2, DNA replicon, RNA replicon, dual-promoter-driven, dual-reporter genes

## Abstract

The severe acute respiratory syndrome coronavirus-2 (SARS-CoV-2, SARS2) remains a great global health threat and demands identification of more effective and SARS2-targeted antiviral drugs, even with successful development of anti-SARS2 vaccines. Viral replicons have proven to be a rapid, safe, and readily scalable platform for high-throughput screening, identification, and evaluation of antiviral drugs against positive-stranded RNA viruses. In the study, we report a unique robust HIV long terminal repeat (LTR)/T7 dual-promoter-driven and dual-reporter firefly luciferase (fLuc) and green fluorescent protein (GFP)-expressing SARS2 replicon. The genomic organization of the replicon was designed with quite a few features that were to ensure the replication fidelity of the replicon, to maximize the expression of the full-length replicon, and to offer the monitoring flexibility of the replicon replication. We showed the success of the construction of the replicon and expression of reporter genes fLuc and GFP and SARS structural N from the replicon DNA or the RNA that was in vitro transcribed from the replicon DNA. We also showed detection of the negative-stranded genomic RNA (gRNA) and subgenomic RNA (sgRNA) intermediates, a hallmark of replication of positive-stranded RNA viruses from the replicon. Lastly, we showed that expression of the reporter genes, N gene, gRNA, and sgRNA from the replicon was sensitive to inhibition by Remdesivir. Taken together, our results support use of the replicon for identification of anti-SARS2 drugs and development of new anti-SARS strategies targeted at the step of virus replication.

## 1. Introduction

The coronavirus disease that emerged in 2019 (COVID-19) is a highly transmissible respiratory disease and caused by SARS-CoV-2 (SARS2) [1,2,3,4]. More than 450 million people have been infected and more than 6 million have died from this infection worldwide since late 2019 [5]. Infected people develop headache, fever, coughing, diarrhea, and pneumonia, with higher mortality in the elderly and those with compromised immune systems, diabetes, and other chronic respiratory and heart diseases [1,6]. Thus, COVID-19 has been a profound global health threat. The method of choice for detection and diagnosis of SARS2 infection is real-time RT-PCR, which is very sensitive and accurate and has successfully been adapted for various human specimens including nasal swabs and sputum [7,8]. Vaccines against SARS2 are effective in shortening the duration of virus shedding and improving clinical outcomes, but do not completely prevent viral shedding and transmission [9,10,11,12]. CRISPR/Cas13b/d targets SARS2 RNA degradation and has been proposed as an antiviral strategy [13,14]. Antiviral drugs Remdesivir and Molnupiravir are also used to treat and prevent COVID-19, but with very modest efficacy [15,16,17,18,19,20]. Moreover, over 30% of the population who were infected with SARS2 and recovered from COVID-19 have experienced long-COVID symptoms [21,22,23,24,25,26]. The continued emergence of SARS2 variants, lack of effective and direct SARS2-targeting drugs, and a large population with long-COVID symptoms make it imperative to develop more effective and SARS2-specific antiviral drugs to treat and prevent the disease.

SARS2 is a member of the coronavirus family and is a single-stranded positive enveloped RNA virus [27,28,29]. The viral genome is about 30 kb nucleotides in length and has a leader cap structure and an untranslated region (UTR) at the 5′ end and an UTR and a poly(A) tail at the 3′ end. Both UTRs form highly specified RNA structures required for viral RNA translation, transcription, and replication. There are 14 open reading frames (ORF) within the viral genome, preceded by transcriptional regulatory sequences (TRS). The two main ORFs are ORF1a and ORF1b. ORF1a encodes a large polyprotein, which is cleaved into nonstructural proteins (NSP) 1–11. ORF1b is derived from the frameshift at the 3′ end of ORF1a and, as a result, encodes another large polyprotein, which is cleaved into NSP1-10 and NSP12-16. All these NSPs make up the replication/transcription complex with distinct functions and are essential for viral RNA replication/transcription, which includes first synthesis of negative-stranded viral genomic RNA (gRNA) and subgenomic RNA (sgRNA), and subsequent synthesis of positive-stranded gRNA and sgRNA. Four structural proteins: spike S, envelope E, membrane M, and nucleocaspid N, and nine accessory proteins: ORF3a/b, 6, 7a/b, 8, 9a/b, and 10 are encoded by their corresponding sgRNA. Newly synthesized gRNA and structural proteins are assembled to form new infectious virions for the new round of infection. Due to the unique features and viral proteins (enzymes) involved, the RNA replication and transcription process affords the best targets to develop direct SARS2-targeting antiviral drugs.

Replicons have been the strategy of choice to screen and identify antiviral drugs for positive-stranded RNA viruses and to study molecular mechanisms of the viral replication process. They are constructed by reverse genetic engineering of partial viral genomes and one or more structural genes so that the viral genome can replicate and persist in cells. Replicons have successfully been constructed in several families of positive-stranded RNA viruses including *picornaviridae* [30], *caliciviridae* [31], *flaviviridae* [32,33,34,35,36,37], and *coronaviridae* [38,39,40,41,42]. A great example is HCV replicons, which have been attributed to successful identification of several direct acting antivirals to treat HCV infections [43,44]. All the replicons lack the envelope gene and other structural genes, and there are no infectious viruses produced from use of the replicons. Thus, replicons represent an ideal platform for identification of anti-SARS2 antiviral drugs and elucidation of molecular mechanisms of SARS2 replication for researchers, particularly for those who do not have access to a research facility of biosafety level 3 or higher required for working with SARS2.

In this study, we created a unique robust SARS2 replicon with dual-promoters HIV long terminal repeat (LTR) and T7 and with dual-reporters luciferase and green fluorescent protein. We also incorporated several other novel features into the design of the replicon, which would together afford the replication fidelity of the replicon, maximized expression of the full-length replicon, and flexible monitoring of the replicon replication. Our findings indicate that the replicon may provide a platform for rapid, sensitive, and safe screening and evaluation of the SARS2 replication inhibitors.

## 2. Materials and Methods

### 2.1. Cells, Transfection, and Remdesivir Treatment

For this study, 293T and Vero E6 were purchased from American Tissue Culture Collection (Manassas, VA, USA) and were maintained in DMEM (Sigma-Aldrich, Burlington, MA, USA) supplied with 10% FBS (Atlanta Biologicals, Flowery, GA, USA) and penicillion/streptomycin in a 37 °C, 5% CO_2_ incubator. Cells were transfected with Lipofectamin 3000 (ThermoFisher Scientific, Waltham, MA, USA) according to the manufacturer’s protocol. For experiments involving Remdesivir treatment, the cells were treated with Remdesivir for 1 h before transfection, continued with Remdesivir treatment for 24 h following cell transfection with DNA or RNA, and harvested for the luciferase reporter gene assay, Western blotting, and RT-PCR analysis. Remdesivir was purchased from Cayman Chemical Company (Ann Arbor, MI, USA) and dissolved in DMSO.

### 2.2. Synthesis of Replicon Fragments and Construction of Recombinant Non-Infectious SARS2 Replicon DNA

The full-length SARS2 DNA replicon is 27,952 nucleotides. It was synthesized in five fragments onto the pMX vector (for F2-5) or pMK vector (for F1/6) and sequenced by ThermoFisher Scientific. The sizes of the fragments were as follows: F2-nt 3583–8945; F3-nt 8942–15,011; F4-nt 15,007–21,092; F5-nt 21,078–24,749; F1/6-nt 1–1642/nt 24,689–27,952. The fragments were designed with either BsaI or SalI restriction sites for subsequent cloning. Fragment F2-5 on pMX were ligated together at the unique BsaI sites using a Golden Gate Assembly kit (New England Biolabs, Ipswich, MA, USA). Specifically, a ligation reaction (20 μL) containing 200 ng each of the four constructs (pMX.F2, 3, 4, or 5), T4 DNA ligase buffer, and Golden Gate Enzyme Mix was set up and incubated at 37 °C for 1 h to obtain the ligated product containing fragment 2–5 of the replicon on the pMX vector (pMX.F2-5). The ligated product was gel-purified, digested with SalI, and gel-purified to obtain fragment F2-5. pMK.F1/6 was digested with SalI and annealed with SalI-digested F2-5 through a pre-designed 50-nucleotide homolog overhang using a Gibson Assembly kit (New England Biolabs). Specifically, a reaction (20 μL) containing fragment F2-5, SalI-digested pMK.F1/6, and Gibson Assembly Master Mix was set up and incubated at 50 °C for 1 h. An aliquot of the reaction was transformed into B10 competent *E. coli* (New England Biolabs). The final replicon DNA construct pMK.F1–6 was verified by PCR with five pairs of primers spanning each of the five adjacent junctions: 5′-aag atc gcc gtg taa gaa ttc cg-3 (nt 3315–3337) and 5′-tgc ccg cgg tta tca tcg tgt t-3′ (nt 3919–3898) for F1/F2; 5′-tgc ata gac ggt gct tta ctt ac-3′ (nt 8915–8937) and 5′- ggt aca aga tca att ggt tgc tc-3′ (nt 9123–9101) for F2/F3; 5′-ggt ggc aaa cct tgt atc aaa g-3′ (nt 14,918–14,939) and 5′-gag gct ata gct tgt aag gtt gc-3′ (nt 15,213–15,191) for F3/F4; 5′-cag ggc tca gaa tat gac tat g-3′ (nt 20,956–20,977) and 5′-gtg tag gtg cct gtg tag gat-3′ (nt 21,223–21,206) for F4/F5; 5′-ctt tgg ggt act gct gtt atg t-3′ (nt 24,533–24,554) and 5′-cat ctc ctt cac ctt cac cag a-3′ (nt 24,793–24,772) for F5/F6.

### 2.3. Purification of the SARS2 Replicon Plasmid DNA pMK.F1–6 and In Vitro RNA Transcription

A single colony was selected from the B10-transformed *E. coli* plate above, inoculated in 2 mL Amp + LB medium, and cultured at room temperature overnight at a shaker speed of 30 rpm. The 2 mL culture was transferred to 100 mL Amp + LB medium and continued to culture for 48 h. The culture was pelleted and suspended in 2 mL of 50 mM glucose, 50 mM Tris, pH 8.0, 10 mM EDTA, pH 8.0, and 50 μg/mL DNase-free RNase A, had added 4 mL of 0.1 M NaOH and 1% SDS, was incubated at room temperature for 3 min, had added 3 mL of 1.5 M potassium acetate, pH 5.5, and was incubated at room temperature for 10 min. Mixing throughout the process had to be extremely gentle to avoid shearing of the large-size plasmid DNA. Then, the mixture was spun at 3000× *g* for 10 min, the clear supernatant was recovered, had added 2 volumes of isopropanol, was incubated at −20 °C for 10 min, and spun at 10,000× *g* for 10 min. The DNA pellet was rinsed with 75% ethanol, suspended in TE buffer containing 50 μg/mL DNase-free RNase A, incubated at room temperature for 30 min, and extracted with phenol/chloroform/isoamyl for 2–3 min. RNase A treatment and phenol/chloroform/isoamyl extraction were repeated two more times. The aqueous phase had added 2 volumes of isopropanol and was spun at 10,000× *g* for 10 min. The DNA pellet was rinsed with 75% ethanol, dried, and suspended in TE as the replicon plasmid pMK.F1–6 DNA. Throughout the process, all mixing steps had to be extremely gentle to avoid shearing of the plasmid DNA. For in vitro transcription, replicon RNA was synthesized using 10 μg replicon DNA/100 μL reaction and a T7 RoboMAX Express large-scale RNA synthesis kit (Promega, Madison, WI, USA) in which a Ribo m^7^G Cap analog (Promega) was included. The reaction was performed at 25 °C for 24 h, treated with RQ1 RNase-Free DNase (Promega) at 37 °C 15 min, and extracted with phenol:chloroform:isoamyl once and chloroform:isoamyl once. The aqueous phase had added 2 volumes of isopropanol and was spun at 10,000× *g* for 10 min. The RNA pellet was rinsed with 75% ethanol, dried, and suspended in TE as replicon RNA.

### 2.4. Luciferase Reporter Gene Assay

The 293T (1.5 × 10^5^ cells/well) and Vero E6 (1.5 × 10^5^ cells/well) were plated in a 24-well plate, transfected with a total of 0.4 µg DNA or 0.3 µg RNA, cultured for 6–72 h, harvested, and washed with PBS. The cells were lyzed and assayed for the luciferase activity using the Firefly Luciferase Assay system (Promega) according to the manufacturer’s instructions, and an Opticomp Luminometer (MGM Instruments, Hamden, CT, USA).

### 2.5. Western Blotting

The 293T (4 × 10^6^ cells) were plated in a 10 cm plate, transfected with a total of 10 µg DNA or 7.5 µg RNA, cultured for 4–72 h, harvested, and washed with PBS. The cells were lyzed in RIPA buffer (50 mM Tris.HCl, pH 7.4, 150 mM NaCl, 1% Triton X-100, 1% sodium deoxycholate, 0.1% SDS, 2 mM PMSF, and 1X protease inhibitor mixture (Roche, Indianapolis, IN, USA), and incubated on ice for 20 min. The whole-cell lysates (40 μg) were run with 10% SDS-PAGE gel, blotted for direct detection of the GFP signal at 488 nm, or blotted against SARS2 nucleocapsid antibody (1:500; BEI Resources, Manassas, VA, USA), followed by ECL visualization (ThermoFisher Scientific). Blots were striped for re-probing against an anti-β-actin antibody (Sigma-Aldrich).

### 2.6. Semi-Quantitative RT-PCR Determination of (+) and (−) Strand SARS2 Replicon Genomic RNA (gRNA) or N Subgenomic RNA (sgRNA)

The 293T (6.5 × 10^5^ cells/well) were plated in a 6-well plate, transfected with a total of 1.5 µg DNA or 1.2 µg RNA, cultured for 24 h, harvested, and washed with PBS. Total RNA was isolated from cells using a Trizol Reagents kit (ThermoFisher) according to the manufacturer’s instructions, treated with RQ1 RNase-free DNase in 1X RQ1 DNase reaction buffer (Promega) at 37 °C 10 min, and extracted with an equal volume of acidic phenol (ThermoFisher Scientific). The aqueous phase had added 2 volumes of isopropanol and was spun at 10,000× *g* for 10 min. The RNA pellet was rinsed with 75% ethanol and suspended in RNase-free water (ThermoFisher Scientific). Primer TRS-L5′ was used to reverse-transcribe (−) strand RNA to cDNA, while primer N3′ was used to reverse-transcribe (+) strand RNA to cDNA. Primer pair N5′/N3′ was used to PCR-amplify the full-length N gene (1260 bp) from both (+) and (−) strand gRNA and sgRNA-derived cDNA. Primer pair TRS-L5′/N3′ was used to PCR amplify both (+) and (−) strand sgRNA-derived cDNA. The sequences of the primers were as follows: TRS-L5′: 5′-atc tct tgt aga tct gtt ctc taa acg aac aaa cta aa-3′ (nt 845–874); N-5′: 5′-atg tct gat aat gga ccc ca-3′ (nt 28,274–29,291); N-3′: 5-tta ggc ctg agt tga gtc ag-3′ (nt 295,534–29,512).

### 2.7. Data Analysis

A two-tailed Student’s *t*-test was performed for all two-way comparisons. All values are expressed as Mean ± SEM. A *p* value less than 0.05 was considered significant, and *p* value less than 0.01 was considered highly significant.

## 3. Results

### 3.1. Design and Construction of the LTR/T7 Dual-Promoter-Driven and GFP/fLuc Dual-Reporter-Expressing SARS2 Replicon

**Figure 1:**
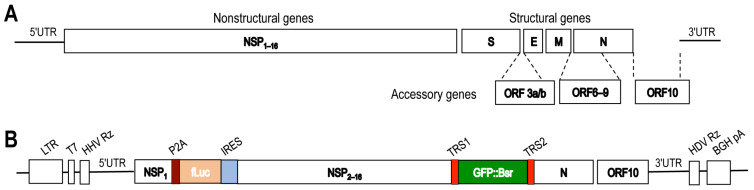
Scheme of SARS2 genome and the SARS2 replicon DNA construct. (**A**). The full-length of SARS2 genome from the Wuhan-Hu-1 isolate (GenBank accession No. NC_045512.2) encodes 5′ untranslated region (UTR), nonstructural proteins NSP1-16, structural proteins S, E, M, and N, accessory proteins ORF3-10, and 3′UTR. (**B**). Several genetic elements were included in the recombinant replicon DNA construct for various purposes. These include HIV long terminal repeat (LTR) promoter, T7 promoter, hammerhead virus ribozyme (HHV Rz) at the 5′ end, porcine teschovirus-1 self-cleaving peptide 2A (P2A) between NSP1 aa1-183 and firefly luciferase, encephalomyocarditis virus internal ribosome entry site (IRES) before NSP2-16, green fluorescence protein-blasticidine (GFP::Bsr) in place of S/E/M, and hepatitis delta virus ribozyme (HDV Rz) and bovine growth hormone polyadenylation signal (BGH pA) at the 3′ end.

Using as the reference the Wuhan-Hu-1 isolate SARS2 genome (GenBank: NC_045512.2, Figure 1A), we designed the two-in-one SARS replicon to have both HIV LTR promoter and T7 promoter at the 5′end (Figure 1B). When co-expressed with HIV Tat protein in cells, the DNA replicon would ensure transcription of the unusually large full-length viral RNA replicon through the LTR promoter [45,46,47,48]. Alternatively, the DNA replicon could be used as the template to synthesize the RNA replicon using a T7 DNA-dependent RNA polymerase-based in vitro transcription kit, which often gave rise to a limited amount of the full-length RNA of this large size. A few other features had been incorporated into the replicon (Table 1). Hammerhead virus ribozyme site (HHV Rz) and Hepatitis Delta virus ribozyme site (HDV Rz) were inserted immediately before 5′ end and after the 3′ end of SARS2, respectively, to allow removal of extra nucleotides at both 5′ and 3′ ends from the nascent RNA transcribed from the replicon DNA either by cellular transcription machinery or the RNA from in vitro transcription, so that an RNA replicon with authentic 5′ and 3′ ends was produced to faithfully recapitulate RNA replication and transcription. For the same reason, NSP1 and ORF10 adjacent to the 5′ end and 3′ end were kept in the replicon design. HDV Rz would also allow direct use of the DNA replicon for in vitro transcription without linearizing the DNA. The firefly luciferase reporter gene (fLuc) was inserted between NSP1 and NSP2-16 to monitor translation and replication/transcription of the replicon, while the GFP::Bsr fusion gene was inserted between NSP16 and N to monitor replication/transcription of the replicon and selection of stable cell replicons. The N gene and its transcriptional regulatory sequence (TRS) were kept for efficient SARS gRNA and N sgRNA replication and N protein expression [41]. The TRS of the S gene was inserted before the GFP::Bsr fusion gene for GFP::Bsr sgRNA replication and GFP::Bsr protein expression. Porcine teschovirus-1 self-cleaving peptide 2A (P2A) was inserted between NSP1 and fLuc to ensure proper processing of NSP1 and fLuc. Encephalomarcarditis virus internal ribozyme entry site (IRES) was inserted before the NSP2-16 gene to facilitate translation of the large polypeptide NSP2-16 from the RNA replicon. Bovine growth hormone polyadenylation signal (BGH pA) was added to the 3′ end to stabilize the RNA.

**Figure 2:**
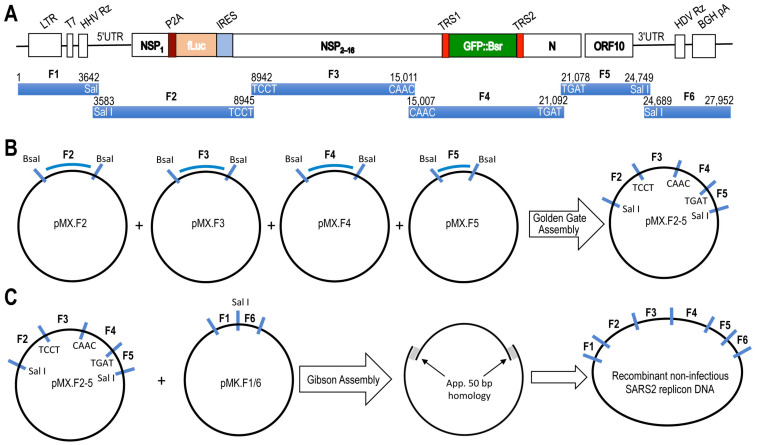
Construction of the SARS2 replicon DNA. (**A**) The full-length of the recombinant SARS2 replicon DNA (27,952 bp) was divided into and synthesized in 5 fragments (F1/F6 and F2-F5) in the backbone of pMX backbone vector (for F2-5) or pMK backbone vector (for F1/6) with approximate nucleotide sequences for BsaI or SalI restriction sites at both 5′ and 3′ end. (**B**) Fragments F2-5 in pMX were ligated to create pMX.F2-5 construct using a Golden Gate Assembly kit. (**C**) pMXF2-5 and pMKF1/6 were digested with SalI and ligated to create the full-length recombinant non-infectious SARS2 replicon DNA construct using a homology recombination-based Gibson Assembly kit.

**Figure 3:**
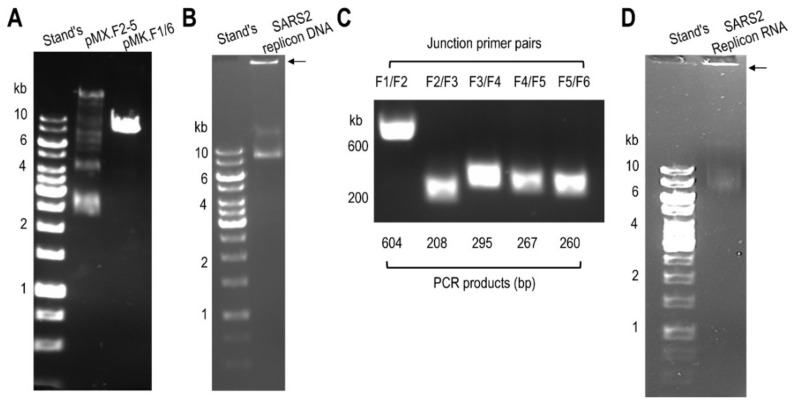
Recombinant SARS2 replicon DNA and its transcribed replicon RNA. (**A**). The intermediate product pMXF2-5 DNA from the Golden Gate Assembly and pMK.F1/6 DNA were confirmed using 0.5% agarose gel electrophoresis. (**B**). The SARS replicon DNA was obtained from pMX.F2-5 and pMK.F1/6 using a Golden Gate Assembly kit and confirmed using 0.5% agarose gel electrophoresis, marked by an arrow. (**C**). The full-length recombinant SARS2 replicon DNA was confirmed by PCR using primer pairs spanning specific junctions between two adjacent DNA fragments. (**D**). SARS2 DNA replicon was used to synthesize SARS2 RNA replicon using an in vitro T7 transcription kit, and the RNA replicon was confirmed using denatured agarose electrophoresis (0.7%), marked by an arrow. Stand’s: DNA size markers.

To construct the two-in-one dual-reporter SARS2 DNA replicon (27,952 nucleotides), five fragments were divided into five fragments (F1/6 and F2, F3, F4, and F5, Figure 2A) and synthesized onto the pMX vector (for F2-5) or pMK vector (for F1/6) with appropriate restriction sites (BasI or SalI) for subsequent cloning and validation by sequencing. F2-5 were first ligated using a Golden Gate Assembly kit (Figure 2B). The intermediate construct pMX.F2-5 was digested with SalI and annealed with SalI-digested pMK.F1/6 using a Gibson Assembly kit (Figure 2C and Figure 3A) to obtain the final DNA construct pMK.F1–6, the DNA replicon (Figure 3B). The DNA replicon was verified by PCR with five pairs of primers spanning each of the five adjacent junctions (Figure 3C). To synthesize the RNA replicon, the DNA replicon was used as the template in an in vitro transcription with inclusion of a Ribo m^7^G Cap analog. The capped RNA replicon was purified, analyzed by denatured agarose gel electrophoresis, and estimated to be the right size (Figure 3D).

### 3.2. Expression of the Reporter Genes and SARS2 N Gene from the LTR/T7 Dual-Promoter-Driven and GFP/fLuc Dual-Reporter-Expressing SARS2 Replicon

**Figure 4:**
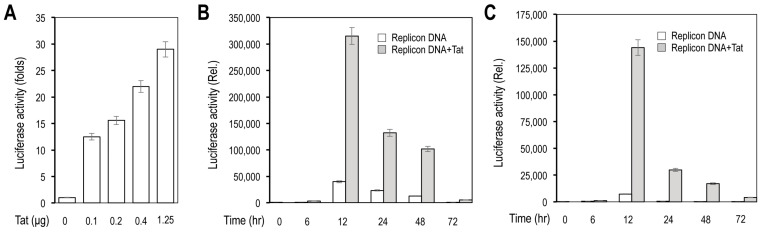
Luciferase reporter gene expression from recombinant SARS2 replicon DNA in response to HIV Tat expression (**A**,**B**). The 293T were plated at a density of 1.5 × 10^5^ cells/well in a 24-well plate, transfected with 0.4 μg SARS2 replicon DNA and an increasing amount of pc3.Tat, cultured for 24 h, and harvested for the luciferase activity assay (**A**), or transfected with 0.4 μg SARS2 replicon DNA and 0.12 μg pc3.Tat, cultured for different lengths of time, and harvested for the luciferase activity assay (**B**,**C**). Vero E6 were transfected with 0.4 μg SARS2 replicon DNA and 0.12 μg pc3.Tat, cultured for different lengths of time, and harvested for the luciferase activity assay. pcDNA3 was used to equalize the total amount of DNA among all transfections. The data were Mean ± SEM and representative of at least three independent experiments. All differences were highly significant compared to Tat (0 μg) (**A**), and compared to Time (0 h), except Time (6 h) and between Replicon DNA and Replicon DNA + Tat (**B**,**C**).

**Figure 5:**
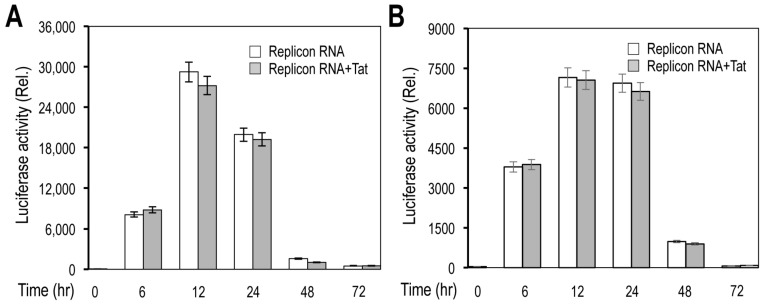
Luciferase reporter gene expression from SARS2 replicon RNA in response to HIV Tat expression. The 293T (**A**) or Vero E6 (**B**) were plated at a density of 1.5 × 10^5^ cells/well for 293T and 1.5 × 10^5^ cells/well for Vero E6 in a 24-well plate, transfected with 0.3 μg SARS2 replicon RNA and 0.1 μg pc3.Tat, cultured for different lengths of time, and harvested for the luciferase activity assay. pcDNA3 was used to equalize the total amount of DNA among all transfections. The data were Mean ± SEM and representative of at least three independent experiments. All differences were highly significant compared to Time (0 h) and insignificant between Replicon RNA and Replicon RNA + Tat.

We then determined the gene expression from the new replicon. The first reporter gene fLuc was inserted between NSP1 and NSP2–16. Thus, the fLuc expression could be used as an indicator of translation of positive-stranded RNA that was either transcribed from the replicon DNA, the in vitro transcribed replicon RNA, or gRNA resulting from replication of these initial transcribed RNA. First, we determined the fLuc expression in cells transfected with the replicon DNA and effects of HIV Tat expression on the fLuc expression. fLuc expression, measured by the fLuc activity, was detected in 293T transfected with the replicon DNA alone and increased in 293T co-transfected with Tat expression plasmid pc3.Tat in a dose-dependent manner (Figure 4A). fLuc expression showed increases up to 12 h and then decreases to the minimal level at 72 h in 293T transfected with the replicon DNA alone, while fLuc expression had similar kinetics but at a significantly higher level at each time point in cells co-transfected with pc3.Tat (Figure 4B). Similar results were obtained in Vero E6 (Figure 4C). We next determined fLuc expression in cells transfected with the in vitro transcribed replicon RNA. fLuc expression showed generally similar kinetics in 293T transfected with the replicon RNA (Figure 5A) and Vero E6 transfected with the replicon RNA (Figure 5B). As expected, Tat co-transfection did not show any effects on fLuc expression in cells transfected with the replicon RNA. Compared to the replicon DNA transfection (Figure 4B,C), the replicon RNA transfection gave rise to a higher level of fLuc expression at 6 h post-transfection and had only relatively slight decreases at 24 h from 12 h (Figure 5A,B).

**Figure 6:**
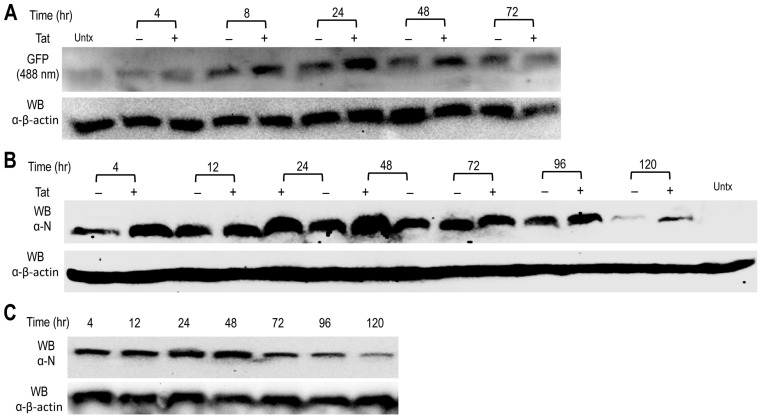
Expression of the GFP reporter gene and SARS2 N (**A**,**B**). The 293T were plated at a density of 4 × 10^6^ cells/plate in a 10 cm plate, transfected with 10 μg SARS2 replicon DNA and 3.3 μg pc3.Tat, cultured for different lengths of time, and harvested for Western blotting and direct imaging of the GFP signal on the blots at 488 nM (**A**), or for Western blotting against an anti-SARS2 N antibody (**B**). Untx: 293T were only transfected with pcDNA3. (**C**). The 293T were at a density of 4 × 10^6^ cells/plate in a 10 cm plate, transfected with 7.5 μg SARS2 replicon RNA, cultured for different lengths of time, and harvested for Western blotting against an anti-SARS2 N antibody. Western blotting against an anti-β-actin antibody was included as the equal loading control. The data were representative of at least three independent experiments.

The second reporter gene GFP was inserted as a fusion protein with Blasticidin S resistance gene (Bsr) between NSP2-16 and N gene, and the GFP gene was preceded with the authentic transcriptional regulatory sequences (TRS) of SARS2 S gene. Thus, GFP expression would represent replication of gRNA and GFP sgRNA and translation of GFP sgRNA. GFP expression was detected in 293T transfected with the replicon DNA at 4 h post-transfection, increased up to 48 h, and then decreased (Figure 6A). Tat co-transfection led to a higher level of GFP expression at each time point of the detection. There was only very dim GFP expression in these cells observed under a fluorescence microscope (data not shown). Only the SARS2 structural gene N and its native TRS configuration were kept in the design of the replicon, as this was required for formation of the replication/transcription complex and replication of coronaviruses [41]. N protein expression would provide additional evidence to support replication of gRNA and sgRNA (N) and translation of N sgRNA. N protein showed similar expression kinetics and response to Tat co-transfection to those of GFP expression (Figure 6B). The N protein was also detected in similar kinetics in 293T transfected with the replicon RNA (Figure 6C). Unlike 293T, Vero E6 showed GFP and N expression by Western blotting and GFP expression under a fluorescence microscope when transfected with the replicon DNA, DNA plus Tat, or the replicon RNA (data not shown).

### 3.3. RNA Transcription and Replication from the LTR/T7 Dual-Promoter-Driven and GFP/fLuc Dual-Reporter-Expressing SARS2 Replicon and its Inhibition by Remdesivir

**Figure 7:**
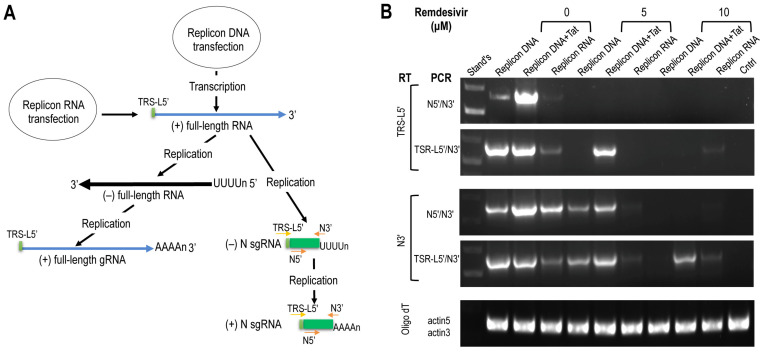
Expression of positive/negative-stranded genomic RNA (gRNA) and N subgenomic RNA (sgRNA) from the SARS2 replicon and its response to Remdesivir. (**A**) Different RT primers (N3′ and LRS-L) in combination with different PCR primers (N5′/N3′ and TSR-L/N3′) were designed to distinguish positive-stranded from negative-stranded gRNA and N sgRNA. RT with N3′, followed by PCR with N5′/N3′ and TSR-L/N3′ represented positive-stranded gRNA and N sgRNA, respectively. RT with TSR-L, followed by PCR with N5′/N3′ and TSR-L/N3′, represented negative-stranded gRNA and N sgRNA, respectively. (**B**) The 293T were plated at a density of 6.5 × 10^5^ cells/well in a 6-well plate, treated with 0, 5, or 10 μM Remdesivir for 1 h, transfected with 1.5 μg SARS2 replicon DNA and 0.5 μg pcDNA3, 1.5 μg SARS2 DNA and 0.5 μg pc3.Tat, or 1.2 μg SARS2 replicon RNA, cultured in the presence of Remdesivir for 24 h, and harvested for RNA isolation. RT was performed using N3′ or TRS-L5′ as the primer and 0.5 μg RNA in a 25 μL reaction. An aliquot RT reaction (2 μL from N3′ RT reaction; 2 μL from TSR-L5′ RT reaction) was used as the template for PCR, with indicated primer pairs. The PCR products were analyzed on 1% agarose gel electrophoresis. RT was performed using 0.1 μg RNA. RT with oligo d(T)_23_ as the RT primer and PCR with β-actin-specific primers were performed and included as the equal loading control. Stand’s: DNA size markers. Ctrl: untransfected cells. The data were representative of at least three independent experiments.

The full-length SARS2 replicon RNA could be derived from *in vitro* transcription of the replicon DNA or transcription of the replicon DNA in cells by cellular transcription machinery, and serve as the template for translation and expression of NSP1-16 and for synthesis of negative-stranded gRNA and sgRNA intermediates, which in turn served as the templates for subsequent synthesis positive-stranded gRNA and sgRNA (Figure 7A). Thus, detection of negative-stranded RNA gRNA and sgRNA was used as an indicator for the replicon replication. We took advantage of an oligonucleotide TRS-L5′ aligned to the TRS leader that was present in the full-length gRNA and sgRNA as the RT primer to synthesize the cDNA [27,49,50,51,52,53], followed by PCR using primers TRS-L5/N3′ for detection of negative-stranded gRNA, or PCR using primers N5′/N3′ for detection of negative-stranded N sgRNA. Total RNA was isolated from 293T transfected with the replicon DNA alone, the replicon DNA and Tat, or the replicon RNA, and then treated with RNase-Free DNase, followed by multiple rounds of acidic phenol extraction to eliminate input DNA contamination, which was verified by the absence of PCR products using the RNA samples and the replicon DNA-specific primers (Figure 3C) as the template (data not shown). RT by TRS-L5′, followed by PCR with N5′/N3′ and TRS-L5′/N3, showed expression of negative-stranded N sgRNA and gRNA in all transfections (upper two panels, Figure 7B). In the meantime, RT was performed using N3′ as the primer, followed by PCR, which also showed expression of positive-stranded N sgRNA and gRNA in all the transfections (middle two panels, Figure 7B). Importantly, Remdesivir, a nucleoside analog and an RNA-dependent RNA polymerase inhibitor that has been used for treatment of COVID-19 patients [54,55,56,57,58], showed significant inhibition at a concentration of 5 μM and even greater inhibition at a concentration of 10 μM of positive- and negative-stranded gRNA and N sgRNA expression.

### 3.4. Inhibition of Gene Expression from the LTR/T7 Dual-Promoter-Driven and GFP/fLuc Dual-Reporter-Expressing SARS2 Replicon by Remdesivir

**Figure 8:**
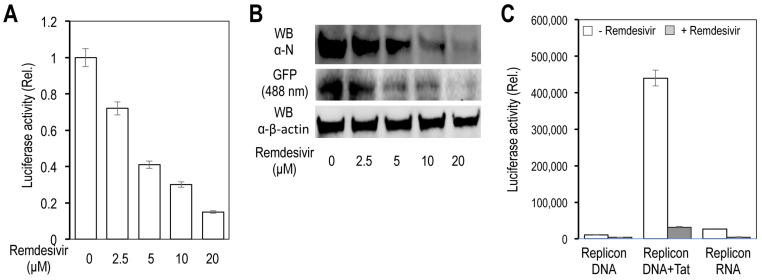
Effects of Remdesivir on gene expression from the SARS2 replicon DNA and RNA (**A**,**B**). The 293T were at a density of 4 × 10^6^ cells/plate in a 10 cm plate, treated with Remdesivir for 1 h, transfected with 10 μg SARS2 replicon DNA, cultured in the presence of Remdesivir for 24 h, and harvested for the luciferase activity assay (**A**), or for Western blotting against an anti-SARS2 N antibody or anti-β-actin antibody, or by direct imaging of the GFP signal at 488 nm (**B**). (**C**). The 293T were at a density of 1.5 × 10^5^ cells/well in a 24-well plate, treated with 10 μM Remdesivir for 1 h, transfected with 0.4 μg SARS2 replicon DNA and 0.12 μg pcDNA3, 0.4 μg SARS2 DNA and 0.12 μg pc3.Tat, or 0.3 μg SARS2 replicon RNA and 0.1 μg yeast tRNA, cultured in the presence of Remdesivir for 24 h, and harvested for the luciferase activity assay. The controls for Remdesivir treatment were DMSO, the solvent for Remdesivir. The data were Mean ± SEM and representative of at least three independent experiments (**A**,**C**) and representative of at least three independent experiments (**B**). All differences were highly significant compared to Remdesivir (0 μM) (**A**) and between ± Remdesivir (**C**).

We also determined effects of Remdesivir on expression of these two reporter genes and SARS N gene from the replicon. The 293T were transfected with the replicon and treated with different concentrations of Remdesivir. Remdesivir inhibited the fLuc gene expression in a concentration-dependent manner (Figure 8A). Expression of GFP and N genes showed similar kinetics of inhibition by Remdesivir (Figure 8B). Compared to the DNA replicon in which Remdesivir (10 μM) inhibited fLuc by 70%, the same concentration of Remdesivir inhibited fLuc from the DNA replicon and Tat by about 15-fold and from the RNA replicon by about 18-fold (Figure 8C).

## 4. Discussion

In the study, we designed, constructed, and characterized a dual-promoter-driven and dual-reporter-expressing SARS2 replicon. Our replicon contained the genomic organization from 5′ end to 3′ end: LTR-T7-HHD Rz-5′UTR-NSP1-P2A-fLuc-IRES-NSP2-16-TRS-GFP::Bsr-TRS-N-ORF10-3′UTR-HDV Rz-BGH pA. Over the past two years, several SARS-CoV-2 replicons have been developed [59,60,61,62,63,64,65,66,67,68]. The general genomic composition of these SARS-CoV-2 replicons includes 5′UTR, ORF1a/1b, a Luc gene or green fluorescence protein (GFP) reporter gene, N, and 3′UTR from 5′ end to 3′ end. However, they differ in how the replicon RNA is produced. Some replicons have a T7 promoter at the 5′ end, and the replicon RNA has to be synthesized in vitro [59,60,61,62,63,64]. Other replicons have the human cytomegalovirus (CMV) immediate-early enhance and promoter at the 5′ end, and the replicon RNA is transcribed from the replicon DNA that is introduced into cells by transfection [65,66,67,68]. There were several main features that still remained unique to our replicon. These included efficient full-length RNA transcription under the HIV LTR promoter and its transactivation by Tat co-expression, production of the replicon RNA with 5′ and 3′ ends that are identical to the native SARS2, fLuc insertion within the NSP genes as the indicator for translation and replication/transcription of the replicon RNA, placement of IRES before NSP2-16 to facilitate NSP2-16 translation and expression, and one cassette 5UTR-NSP1 at the 5′ end and one cassette ORF10-3′UTR at the 3′ end to maintain the native RNA secondary structure for RNA translation, replication, and transcription.

Our replicon, when transfected into cells in the form of DNA, or RNA that was transcribed from the DNA, showed successful expression of reporter genes fLuc, GFP, and SARS2 structural gene N in 293T and Vero E6. We then demonstrated expression of negative-stranded gRNA and SARS2 N sgRNA, which provided additional evidence to support expression and replication of the replicon in these cells. Lastly, we demonstrated that an RNA-dependent RNA polymerase inhibitor that has been used to treat SARS2 infection inhibited expression of reporter genes fLuc, GFP, and SARS2 structural gene N and negative- and positive-stranded gRNA and SARS2 N sgRNA in 293T and Vero E6. We also included two other cell lines, Hela and Huh7, in the study and obtained similar results (data not shown). All the findings together support the notion that the new replicon could be used as a surrogate system for screening and identifying anti-SARS2 antiviral drugs and for studying the molecular mechanisms of the host and viral control of SARS2 replication and transcription.

The reporter gene GFP was introduced as an indicator to monitor RNA replication/transcription from the replicon. However, to our surprise we were only able to detect GFP expression on Western blots and only a very weak signal under the fluorescence microscope. This phenomenon did appear to be cell type-dependent, as brighter GFP was detected in Vero E6 than 293T. This overall lower level of GFP expression may be due to conformational changes of GFP in the GFP-Bsr fusion protein. It is important to point out that we attempted to establish stable cell replicons, namely, the cells stably expressing the SARS2 replicon, by single cell or bulking cloning with inclusion of Bsr in the culture medium and passages, quite a few times, using different cell lines, and using both the replicon DNA transfection and the replicon RNA transfection. However, we were not successful. Thus, we performed all our studies using the transient replicon by transfection. One likely explanation is the cytotoxicity resulting from a higher level of expression of one of the NSP proteins or structural N protein from our DNA or RNA replicon, which may prevent us from obtaining the stable cell replicons. The other possibility is the instability of the engineered replicon so that the replicon RNA loses the ability to self-replicate in a long-term and sustainable manner. Nevertheless, of note is that among all ten published SARS2 replicons thus far, only two SARS2 replicons lead to creation of stable replicon cells [59,68], and the other eight all appear to be transient replicons [60,61,62,63,64,65,66,67]. Further understanding of the design and organizational differences between these two groups of SARS2 replicons may lead to identification of viral and host factors necessary for SARS2 replication as well as help construct better SARS2 replicons for anti-SARS2 drug screening and evaluation.

## Figures and Tables

**Table 1:** Details of all the features of the SARS2 replicon.

Feature	Abbreviation	Location	Size (bp)	Function
HIV-1 LTR	LTR	1–713	713	To facilitate expression of long RNA transcript when transfected with HIV Tat
T7 promoter	T7	722–740	19	To synthesize viral RNA by in vitro transcription
Hammerhead virus ribozyme	HHV Rz	740–799	59	To produce the native 5′ end of SARS2 viral RNA genome
SARS2 5′ untranslated region	5′UTR	800–1064	265	To maintain the regulatory element for SARS2 replication
SARS2 nonstructural protein 1	NSP1	1065–1604	549	To encode NSP1
Porcine teschovirus-1 self-cleaving peptide 2A	P2A	1611–1671	66	To cleave NSP1-fLuc fusion protein to ensure proper fLuc expression
Firefly luciferase	fLuc	1677–3329	1653	To monitor translation from and replication of SARS2 RNA
Encephalomarcarditis virus internal ribozyme entry site	IRES	3330–3910	581	To facilitate translation of the long transcript of SARS2 NSP2-16
SARS2 nonstrctural protein 2-16	NSP2-16	3917–24,666	20750	To encode nonstructural SARS proteins NSP2-16 for replication
Transcriptional regulatory sequence 1	TRS1	24,675–24,681	7	To maintain the authentic regulatory element for GFP::Bsr expression
GFP-blasticidin S resistance fusion protein	GFP::Bsr	24,682–25,998	1317	To select stable SARS2 replicon and monitor SARS2 replicon replication
Transcriptional regulatory sequence 2	TRS2	25,999–26,102	14	To maintain the authentic regulatory element for SARS2 nucleocapsid expression
SARS2 nucleocapsid protein	N	26,013–27,272	1260	To encode SARS2 nucleocapsid and monitor SARS2 replicon replication
SARS2 ORF10-3′ untraslated region	ORF10-3′UTR	27,373–27,642	370	To maintain the regulatory element integrity for SARS2 replication
Hepatitis delta virus ribozyme	HDV Rz	27,643–27,721	79	To produce the native 3′ end of SARS2 viral RNA genome
Bovine growth hormone polyadenylation signal	BGH pA	27,728–27,952	225	To stabilize the RNA transcript

## Data Availability

All data and information reported in this article will be shared by the corresponding author upon request.

## References

[1] Zhang G., Zhang J., Wang B., Zhu X., Wang Q., Qiu S. (2020). Analysis of clinical characteristics and laboratory findings of 95 cases of 2019 novel coronavirus pneumonia in Wuhan, China: A retrospective analysis. Respir. Res..

[2] Tan W., Zhao X., Ma X., Wang W., Niu P., Xu W., Gao G.F., Wu G. (2020). A Novel Coronavirus Genome Identified in a Cluster of Pneumonia Cases—Wuhan, China 2019–2020. China CDC Wkly..

[3] Meng H., Xiong R., He R., Lin W., Hao B., Zhang L., Lu Z., Shen X., Fan T., Jiang W. (2020). CT imaging and clinical course of asymptomatic cases with COVID-19 pneumonia at admission in Wuhan, China. J. Infect..

[4] Chen N., Zhou M., Dong X., Qu J., Gong F., Han Y., Qiu Y., Wang J., Liu Y., Wei Y. (2020). Epidemiological and clinical characteristics of 99 cases of 2019 novel coronavirus pneumonia in Wuhan, China: A descriptive study. Lancet.

[5] (2022). World Health Organization Coronavirus (COVID-19) Dashboard. http://COVID19.WHO.int.

[6] Zhou P., Yang X.L., Wang X.G., Hu B., Zhang L., Zhang W., Si H.R., Zhu Y., Li B., Huang C.L. (2020). A pneumonia outbreak associated with a new coronavirus of probable bat origin. Nature.

[7] Pu R., Liu S., Ren X., Shi D., Ba Y., Huo Y., Zhang W., Ma L., Liu Y., Yang Y. (2022). The screening value of RT-LAMP and RT-PCR in the diagnosis of COVID-19: Systematic review and meta-analysis. J. Virol. Methods.

[8] Ionescu M.A. (2022). COVID-19 skin lesions are rarely positive at RT-PCR test: The macrophage activation with vascular impact and SARS-CoV-2-induced cytokine storm. Int. J. Dermatol..

[9] Szabo G.T., Mahiny A.J., Vlatkovic I. (2022). COVID-19 mRNA vaccines: Platforms and current developments. Mol. Ther..

[10] Rahman M.M., Masum M.H.U., Wajed S., Talukder A. (2022). A comprehensive review on COVID-19 vaccines: Development, effectiveness, adverse effects, distribution and challenges. Virusdisease.

[11] Jensen A., Stromme M., Moyassari S., Chadha A.S., Tartaglia M.C., Szoeke C., Ferretti M.T. (2022). COVID-19 vaccines: Considering sex differences in efficacy and safety. Contemp. Clin. Trials.

[12] Huang Z., Su Y., Zhang T., Xia N. (2022). A review of the safety and efficacy of current COVID-19 vaccines. Front. Med..

[13] Abbott T.R., Dhamdhere G., Liu Y., Lin X., Goudy L., Zeng L., Chemparathy A., Chmura S., Heaton N.S., Debs R. (2020). Development of CRISPR as an Antiviral Strategy to Combat SARS-CoV-2 and Influenza. Cell.

[14] Fareh M., Zhao W., Hu W., Casan J.M.L., Kumar A., Symons J., Zerbato J.M., Fong D., Voskoboinik I., Ekert P.G. (2021). Reprogrammed CRISPR-Cas13b suppresses SARS-CoV-2 replication and circumvents its mutational escape through mismatch tolerance. Nat. Commun..

[15] Vitiello A., Ferrara F. (2022). Association and pharmacological synergism of the triple drug therapy baricitinib/remdesivir/rhACE2 for the management of COVID-19 infection. Naunyn Schmiedeberg’s Arch. Pharmacol..

[16] Pagliano P., Sellitto C., Scarpati G., Ascione T., Conti V., Franci G., Piazza O., Filippelli A. (2021). An overview of the preclinical discovery and development of remdesivir for the treatment of coronavirus disease 2019 (COVID-19). Expert Opin. Drug Discov..

[17] Kaka A.S., MacDonald R., Linskens E.J., Langsetmo L., Vela K., Duan-Porter W., Wilt T.J. (2022). Major Update 2: Remdesivir for Adults With COVID-19: A Living Systematic Review and Meta-analysis for the American College of Physicians Practice Points. Ann. Intern. Med..

[18] Angamo M.T., Mohammed M.A., Peterson G.M. (2022). Efficacy and safety of remdesivir in hospitalised COVID-19 patients: A systematic review and meta-analysis. Infection.

[19] Pourkarim F., Pourtaghi-Anvarian S., Rezaee H. (2022). Molnupiravir: A new candidate for COVID-19 treatment. Pharmacol. Res. Perspect..

[20] Khiali S., Khani E., Rouy S.B., Entezari-Maleki T. (2022). Comprehensive review on molnupiravir in COVID-19: A novel promising antiviral to combat the pandemic. Future Microbiol..

[21] Thye A.Y., Law J.W., Tan L.T., Pusparajah P., Ser H.L., Thurairajasingam S., Letchumanan V., Lee L.H. (2022). Psychological Symptoms in COVID-19 Patients: Insights into Pathophysiology and Risk Factors of Long COVID-19. Biology.

[22] Thallapureddy K., Thallapureddy K., Zerda E., Suresh N., Kamat D., Rajasekaran K., Moreira A. (2022). Long-Term Complications of COVID-19 Infection in Adolescents and Children. Curr. Pediatr. Rep..

[23] Mehandru S., Merad M. (2022). Pathological sequelae of long-haul COVID. Nat. Immunol..

[24] Joshee S., Vatti N., Chang C. (2022). Long-Term Effects of COVID-19. Mayo Clin. Proc..

[25] Han Q., Zheng B., Daines L., Sheikh A. (2022). Long-Term Sequelae of COVID-19: A Systematic Review and Meta-Analysis of One-Year Follow-Up Studies on Post-COVID Symptoms. Pathogens.

[26] Desai A.D., Lavelle M., Boursiquot B.C., Wan E.Y. (2022). Long-term complications of COVID-19. Am. J. Physiol. Cell Physiol..

[27] Kim D., Lee J.Y., Yang J.S., Kim J.W., Kim V.N., Chang H. (2020). The Architecture of SARS-CoV-2 Transcriptome. Cell.

[28] Snijder E.J., Decroly E., Ziebuhr J. (2016). The Nonstructural Proteins Directing Coronavirus RNA Synthesis and Processing. Adv. Virus Res..

[29] Sola I., Almazan F., Zuniga S., Enjuanes L. (2015). Continuous and Discontinuous RNA Synthesis in Coronaviruses. Annu. Rev. Virol..

[30] Kaplan G., Racaniello V.R. (1988). Construction and characterization of poliovirus subgenomic replicons. J. Virol..

[31] Thumfart J.O., Meyers G. (2002). Feline calicivirus: Recovery of wild-type and recombinant viruses after transfection of cRNA or cDNA constructs. J. Virol..

[32] Liljestrom P., Garoff H. (1991). A new generation of animal cell expression vectors based on the Semliki Forest virus replicon. Biotechnology.

[33] Khromykh A.A., Westaway E.G. (1997). Subgenomic replicons of the flavivirus Kunjin: Construction and applications. J. Virol..

[34] Lohmann V., Korner F., Koch J., Herian U., Theilmann L., Bartenschlager R. (1999). Replication of subgenomic hepatitis C virus RNAs in a hepatoma cell line. Science.

[35] Behrens S.E., Grassmann C.W., Thiel H.J., Meyers G., Tautz N. (1998). Characterization of an autonomous subgenomic pestivirus RNA replicon. J. Virol..

[36] Pang X., Zhang M., Dayton A.I. (2001). Development of Dengue virus type 2 replicons capable of prolonged expression in host cells. BMC Microbiol..

[37] Shi P.Y., Tilgner M., Lo M.K. (2002). Construction and characterization of subgenomic replicons of New York strain of West Nile virus. Virology.

[38] Hertzig T., Scandella E., Schelle B., Ziebuhr J., Siddell S.G., Ludewig B., Thiel V. (2004). Rapid identification of coronavirus replicase inhibitors using a selectable replicon RNA. J. Gen. Virol..

[39] Ge F., Luo Y., Liew P.X., Hung E. (2007). Derivation of a novel SARS-coronavirus replicon cell line and its application for anti-SARS drug screening. Virology.

[40] Ge F., Xiong S., Lin F.S., Zhang Z.P., Zhang X.E. (2008). High-throughput assay using a GFP-expressing replicon for SARS-CoV drug discovery. Antivir. Res..

[41] Almazan F., Galan C., Enjuanes L. (2004). The nucleoprotein is required for efficient coronavirus genome replication. J. Virol..

[42] Wang J.M., Wang L.F., Shi Z.L. (2008). Construction of a non-infectious SARS coronavirus replicon for application in drug screening and analysis of viral protein function. Biochem. Biophys. Res. Commun..

[43] Bartenschlager R. (2002). Hepatitis C virus replicons: Potential role for drug development. Nat. Rev. Drug Discov..

[44] Randall G., Rice C.M. (2001). Hepatitis C virus cell culture replication systems: Their potential use for the development of antiviral therapies. Curr. Opin. Infect. Dis..

[45] Berkhout B., Gatignol A., Rabson A.B., Jeang K.T. (1990). TAR-independent activation of the HIV-1 LTR: Evidence that tat requires specific regions of the promoter. Cell.

[46] Jakobovits A., Rosenthal A., Capon D.J. (1990). Trans-activation of HIV-1 LTR-directed gene expression by tat requires protein kinase C. EMBO J..

[47] Jeang K.T., Shank P.R., Kumar A. (1988). Transcriptional activation of homologous viral long terminal repeats by the human immunodeficiency virus type 1 or the human T-cell leukemia virus type I tat proteins occurs in the absence of de novo protein synthesis. Proc. Natl. Acad. Sci. USA.

[48] Selby M.J., Bain E.S., Luciw P.A., Peterlin B.M. (1989). Structure, sequence, and position of the stem-loop in tar determine transcriptional elongation by tat through the HIV-1 long terminal repeat. Genes Dev..

[49] Chang J.J., Rawlinson D., Pitt M.E., Taiaroa G., Gleeson J., Zhou C., Mordant F.L., De Paoli-Iseppi R., Caly L., Purcell D.F.J. (2021). Transcriptional and epi-transcriptional dynamics of SARS-CoV-2 during cellular infection. Cell Rep..

[50] Davidson A.D., Williamson M.K., Lewis S., Shoemark D., Carroll M.W., Heesom K.J., Zambon M., Ellis J., Lewis P.A., Hiscox J.A. (2020). Characterisation of the transcriptome and proteome of SARS-CoV-2 reveals a cell passage induced in-frame deletion of the furin-like cleavage site from the spike glycoprotein. Genome Med..

[51] V’Kovski P., Kratzel A., Steiner S., Stalder H., Thiel V. (2021). Coronavirus biology and replication: Implications for SARS-CoV-2. Nat. Rev. Microbiol..

[52] Wang D., Jiang A., Feng J., Li G., Guo D., Sajid M., Wu K., Zhang Q., Ponty Y., Will S. (2021). The SARS-CoV-2 subgenome landscape and its novel regulatory features. Mol. Cell.

[53] Yang Y., Yan W., Hall A.B., Jiang X. (2021). Characterizing Transcriptional Regulatory Sequences in Coronaviruses and Their Role in Recombination. Mol. Biol. Evol..

[54] Beigel J.H., Tomashek K.M., Dodd L.E., Mehta A.K., Zingman B.S., Kalil A.C., Hohmann E., Chu H.Y., Luetkemeyer A., Kline S. (2020). Remdesivir for the Treatment of Covid-19—Final Report. N. Engl. J. Med..

[55] Gordon C.J., Tchesnokov E.P., Feng J.Y., Porter D.P., Gotte M. (2020). The antiviral compound remdesivir potently inhibits RNA-dependent RNA polymerase from Middle East respiratory syndrome coronavirus. J. Biol. Chem..

[56] Grein J., Ohmagari N., Shin D., Diaz G., Asperges E., Castagna A., Feldt T., Green G., Green M.L., Lescure F.X. (2020). Compassionate Use of Remdesivir for Patients with Severe Covid-19. N. Engl. J. Med..

[57] Kokic G., Hillen H.S., Tegunov D., Dienemann C., Seitz F., Schmitzova J., Farnung L., Siewert A., Hobartner C., Cramer P. (2021). Mechanism of SARS-CoV-2 polymerase stalling by remdesivir. Nat. Commun..

[58] Wang Q., Wu J., Wang H., Gao Y., Liu Q., Mu A., Ji W., Yan L., Zhu Y., Zhu C. (2020). Structural Basis for RNA Replication by the SARS-CoV-2 Polymerase. Cell.

[59] Liu S., Chou C.K., Wu W.W., Luan B., Wang T.T. (2022). Stable Cell Clones Harboring Self-Replicating SARS-CoV-2 RNAs for Drug Screen. J. Virol..

[60] Ricardo-Lax I., Luna J.M., Thao T.T.N., Le Pen J., Yu Y., Hoffmann H.H., Schneider W.M., Razooky B.S., Fernandez-Martinez J., Schmidt F. (2021). Replication and single-cycle delivery of SARS-CoV-2 replicons. Science.

[61] Zhang Q.Y., Deng C.L., Liu J., Li J.Q., Zhang H.Q., Li N., Zhang Y.N., Li X.D., Zhang B., Xu Y. (2021). SARS-CoV-2 replicon for high-throughput antiviral screening. J. Gen. Virol..

[62] Kotaki T., Xie X., Shi P.Y., Kameoka M. (2021). A PCR amplicon-based SARS-CoV-2 replicon for antiviral evaluation. Sci. Rep..

[63] He X., Quan S., Xu M., Rodriguez S., Goh S.L., Wei J., Fridman A., Koeplinger K.A., Carroll S.S., Grobler J.A. (2021). Generation of SARS-CoV-2 reporter replicon for high-throughput antiviral screening and testing. Proc. Natl. Acad. Sci. USA.

[64] Zhang H., Fischer D.K., Shuda M., Moore P.S., Gao S.J., Ambrose Z., Guo H. (2022). Construction and characterization of two SARS-CoV-2 minigenome replicon systems. J. Med. Virol..

[65] Nguyen H.T., Falzarano D., Gerdts V., Liu Q. (2021). Construction of a Noninfectious SARS-CoV-2 Replicon for Antiviral-Drug Testing and Gene Function Studies. J. Virol..

[66] Wang B., Zhang C., Lei X., Ren L., Zhao Z., Wang J., Huang H. (2021). Construction of Non-infectious SARS-CoV-2 Replicons and Their Application in Drug Evaluation. Virol. Sin..

[67] Luo Y., Yu F., Zhou M., Liu Y., Xia B., Zhang X., Liu J., Zhang J., Du Y., Li R. (2021). Engineering a Reliable and Convenient SARS-CoV-2 Replicon System for Analysis of Viral RNA Synthesis and Screening of Antiviral Inhibitors. mBio.

[68] Tanaka T., Saito A., Suzuki T., Miyamoto Y., Takayama K., Okamoto T., Moriishi K. (2022). Establishment of a stable SARS-CoV-2 replicon system for application in high-throughput screening. Antivir. Res..

